# Identification and NMR-based structural characterization of the functional domain of EPC3, a virulence effector of the phytopathogenic fungus *Colletotrichum orbiculare*

**DOI:** 10.3389/fpls.2025.1691993

**Published:** 2025-10-10

**Authors:** Zhe Xu, Suthitar Singkaravanit-Ogawa, Yoshitaka Takano, Shinya Ohki

**Affiliations:** ^1^ Center for Nano Materials and Technology, Japan Advanced Institute of Science and Technology (JAIST), Nomi, Japan; ^2^ Graduate School of Agriculture, Kyoto University, Kyoto, Japan

**Keywords:** *Colletotrichum orbiculare*, effector, EPC3, functional domain, NMR

## Abstract

Plant pathogens secrete various effector proteins to induce infections in their host plants. Understanding the molecular basis of plant pathogen effectors is important for improving agricultural productivity, plant health, and sustainability. However, this remains a significant challenge. EPC3 (EPC; Effector Protein for Cucurbit infection) is a recently discovered effector involved in the virulence of the cucurbit anthracnose fungus *Colletotrichum orbiculare* on host plants, although the structure-function relationship is unknown. Here, we report that the N-terminal half domain of EPC3 is responsible for its function. We determined the solution nuclear magnetic resonance (NMR) structure and dynamic properties of this functional domain. The structure containing three disulfide (SS) bonds is composed of five β-strands. The molecule was rigid except for the loop regions connecting β-strands. The structural properties were compared with those of other structurally similar effectors to deduce the potential residues responsible for this function. Furthermore, mutation experiments demonstrated the importance of intramolecular disulfide bonds in maintaining the structural integrity of EPC3. Our results provided insights into the molecular characteristics of EPC3 and a basis for future structure-guided functional studies.

## 1 Introduction

Phytopathogens and plants engage in a dynamic co-evolutionary conflict, driving the emergence of complex infection strategies and robust immune responses. To establish an infection, phytopathogens deploy a diverse array of secreted proteins known as effectors. Effectors exhibit intrinsic biochemical functions and are thought to manipulate host immune responses. Plants have evolved sophisticated immune systems to counter pathogen attacks. Plant immunity is broadly classified based on the type of receptor involved in its activation. Pattern recognition receptors (PRRs), which are localized on the cell surface, recognize conserved microbial molecules, known as microbe-associated molecular patterns (MAMPs), thereby initiating pattern-triggered immunity (PTI). In addition, intracellular nucleotide-binding leucine-rich repeat (NLR) receptors activate effector-triggered immunity (ETI) by detecting pathogen-secreted effectors that interfere with host immunity. In both PTI and ETI, plants exhibit various responses including the activation of mitogen-activated protein kinases (MAPKs), elevation of cytosolic Ca²^+^ concentration, and production of reactive oxygen species (ROS) (Bigeard et al., 2015).

Despite their biological and agricultural importance, the molecular mechanisms underlying pathogen invasion remain poorly understood owing to the extensive diversity of effectors. This diversity poses significant challenges for structural and functional characterization. Identifying and characterizing core effectors that play indispensable roles in virulence are essential for advancing our understanding of plant–pathogen interactions (Win et al., 2012). To date, the genome sequences of many plant pathogens have been analyzed and are available in various public databases, providing valuable insights into a vast number of effector candidates with known signal peptides for secretion. However, information regarding the core effectors that play crucial roles in host infections is limited.

For instance, it was recently reported that *in planta* RNA-seq analyses of the cucurbit anthracnose fungus *Colletotrichum orbiculare* on cucumber identified effector candidates associated with cucurbit infection. Subsequent targeted gene disruption of these candidates revealed that *EPC1*, *EPC2*, *EPC3*, and *EPC4* genes (EPC; *Effector Proteins for Cucurbit infection*) produce key effectors (Inoue et al., 2023). Interestingly, EPC3 is a homolog of SIX6, an effector of the soil-borne fungal plant pathogen *Fusarium oxysporum* (Inoue et al., 2023; Gawehns et al., 2014a). It has been previously suggested that *SIX6* is involved in the virulence of cucurbit-infecting pathotypes, including *F. oxysporum* f. sp. *radicis-cucumerinum* and f. sp. *niveum*, on multiple cucurbit hosts (Gawehns et al., 2014b; van Dam et al., 2017). Moreover, SIX6 in *F. oxysporum* f. sp. *lycopersici* is required for full virulence in tomatoes (Gawehns et al., 2014a), suggesting its role in the pathogenicity in both Cucurbitaceae and Solanaceae hosts. The widespread occurrence and essential role of SIX6 prompted further functional and structural investigations of its conserved homolog, EPC3.

To gain deeper insight into EPC3, we adopted an integrated approach that combines functional and structural analyses. In this study, we report that the N-terminal domain of EPC3 is responsible for its function. Based on these findings, we focused on elucidating the three-dimensional structure and dynamics of this functional fragment. Furthermore, we investigated the role of three specific disulfide bonds in this EPC3 fragment by analyzing a variant lacking all disulfide bonds to assess their contribution to the protein structure.

## 2 Materials and methods

### 2.1 Design of the EPC3 fragments

The secondary and tertiary structures of EPC3 were predicted using Jpred4 (Drozdetskiy et al., 2015) and AlphaFold2 (Jumper et al., 2021), respectively. Based on these predictions, four types of EPC3 fragments were designed for activity measurements.

### 2.2 ROS assay of EPC3 and its fragments via transient expression in *N. benthamiana*

To perform ROS assays in *N. benthamiana*, EPC3 and its truncated fragments EPC3-ND, EPC3-CD, and EPC3-NCD were fused with GFP. Each fragment was amplified using the corresponding forward (F) and reverse (R) primers and the amplified product was cloned into the *Eco*RI site of the binary vector pBICP35:GFP (Irieda et al., 2019) under the control of the CaMV 35S promoter using an In-Fusion cloning system (Clontech, TaKaRa). All *GFP* fusion genes contained a 5×glycine linker. Primers used for plasmid construction are listed in **Supplementary Table S1**. All expression plasmids were transformed into *Agrobacterium tumefaciens* strain GV3101 by electroporation. Transformants were cultured overnight in yeast extract peptone broth supplemented with kanamycin, rifampicin, and gentamicin (each at 50 μg/mL). Bacterial cells were pelleted by centrifugation and resuspended in infiltration buffer composed of 5 g/L Murashige and Skoog salts, 1.95 g/L MES, 20 g/L sucrose, and 200 μM acetosyringone (pH 5.6). The suspension was adjusted to a final OD_600_ of 0.3 and incubated at room temperature for 1 h before infiltration into *N. benthamiana* leaves.

To measure flg22-triggered ROS generation, leaves were harvested 2 days post-infiltration. Leaf discs (5 mm diameter) were floated overnight in sterile distilled water in the dark. On the next day, individual discs were transferred into wells of a 96-well microplate containing 50 μL of distilled water, followed by the addition of 50 μL of luminol-based detection solution. The assay solution consisted of 400 μM luminol (FUJIFILM Wako Pure Chemical Corporation; 127-02581), 20 μg/mL horseradish peroxidase (Sigma–Aldrich; P6782), and 0.5 μM flg22 peptide (Invitrogen). Luminescence was detected in relative light units (RLUs) over a 60-minute period using a Luminoskan Ascent 2.1 luminometer (Thermo Fisher Scientific).

To determine the expression of EPC3 and its truncated derivatives in *N. benthamiana*, total protein was extracted from agro-infiltrated leaves at 2 days post-infiltration. Leaf tissue was homogenized in extraction buffer containing 50 mM Tris-HCl (pH 7.4), 150 mM NaCl, 5% (v/v) glycerol, 0.5% (v/v) Triton X-100, and a protease inhibitor cocktail (Roche, 11836170001). The homogenates were centrifuged at 20,000 × g for 10 min to remove insoluble debris. The protein lysates were separated on 12.5% SDS-PAGE gels and subjected to immunoblot analysis. EPC3 and its truncated fusion protein derivatives were detected using an anti-GFP monoclonal antibody (GFP [B-2]; Santa Cruz Biotechnology), followed by incubation with an HRP-conjugated anti-mouse IgG secondary antibody (Cell Signaling Technology, 7076). BlueStar Prestained Protein Marker (FastGene, Cat. No. NE-MWP03-8) was used as a protein marker.

### 2.3 Expression of EPC3 N-terminal fragments in *E. coli*

Codon usage of the DNA encoding the N-terminal fragment of EPC3 (EPC3-ND; residues 44–111) and its mutant lacking all Cys residues (All-S) was optimized for expression in *E. coli*. Each DNA fragment was synthesized (GenScript, Japan) and inserted into the NcoI/XhoI site of the pET-32b (+) vector (Merck, Germany). These systems were designed to express an individual fusion protein containing an N-terminal Trx tag, followed by a six-histidine tag conjugated to a Tobacco Etch Virus (TEV) cleavage site. Subsequently, each plasmid was transformed into the *E. coli* strain Rosetta-gami™ B (DE3)/pLysS (Merck, Germany).

Initially, transformed *E. coli* cells were cultured on Luria-Bertani (LB) agar plates containing 100 μg/mL ampicillin at 37°C overnight. Next, several single colonies were randomly selected, and each was inoculated into 3 mL of LB broth containing 100 μg/mL ampicillin. The cultures were then incubated at 37°C for approximately 4 h. When the OD_600_ reached approximately 0.6, IPTG (1 M stock solution) was added to the culture medium at a final concentration of 0.1 mM. After IPTG induction, the cells were further incubated at 37°C for 3 h. Following incubation, the cells were harvested by centrifugation at 6,000 × g for 20 min at 4°C using a centrifuge (TOMY High-Speed Refrigerated Centrifuge SRX-201). A small proportion of the collected cells was resuspended in 25 mM Tris-HCl buffer (pH 8.0) and sonicated. After sonication, the supernatant was separated from the pellet by centrifugation. SDS-PAGE was subsequently employed to check whether the target proteins appeared in the soluble fraction.

### 2.4 Purification of EPC3-ND and All-S mutant

The harvested cells expressing EPC3-ND were pelleted by a centrifuge at 6,000 × g for 20 min at 4°C (TOMY High-Speed Refrigerated Centrifuge SRX-201). The pelleted cells were resuspended in 80 mL of Tris-HCl buffer (25 mM and pH 8.0) at room temperature. The resuspended cells were lysed by sonication using an ultrasonic disruptor (UD-201, TOMY). The lysed cell suspension was centrifuged at 29,000 × g for 20 min at 4°C in a centrifuge to collect the soluble fraction. The clarified supernatant was applied to an Ni^2+^-NTA affinity chromatography column. After sample loading, the column was equilibrated and washed with Ni^2+^-NTA buffer without imidazole to remove non-specifically bound proteins. Elution was performed by increasing the imidazole concentration in stepwise gradients of 20, 50, 100, 250, 500, and 1,000 mM. The collected fractions were analyzed using SDS-PAGE to identify the fractions containing the target proteins.

The cell pellet expressing All-S was resuspended in 80 mL of 25 mM Tris-HCl buffer (pH 8.0) and washed by centrifugation at 6,000 × g for 20 min at 4°C using an SRX-201 High-Speed Refrigerated Centrifuge (TOMY). The washed cells were resuspended in 80 mL of 25 mM Tris-HCl (pH 8.0) supplemented with 1mM PMSF and 2.5 mg/mL egg white lysozyme (FUJIFILM Wako, Japan). The suspension was incubated at 37°C with shaking for 1 h to facilitate bacteriolysis. SDS-PAGE showed both the supernatant and precipitate containing the tagged samples. The lysate for All-S was subjected to centrifugation at 29,000 × g for 20 min at 4°C (SRX-201) to separate the supernatant and precipitate. Polyethyleneimine (PEI) was added to the supernatant at a final concentration of 0.5%, and the mixture was incubated on ice for 30 min to precipitate excess nucleic acids. The mixture was centrifuged again under identical conditions, and the resulting supernatant was collected. This sample was purified by Ni²^+^-NTA affinity chromatography using the exact procedure established for EPC3-ND. The precipitate for All-S was resuspended in 8 M urea and 25 mM Tris-HCl (pH 8.0) and incubated at 4°C overnight with shaking to solubilize the denatured proteins completely. After incubation, the sample was subjected to centrifugation at 29,000 × g for 20 min at 4°C (SRX-201) to separate the supernatant and precipitate, and the resulting supernatant was collected. The solution was loaded onto the Ni²^+^-NTA column and washed with Ni²^+^-NTA buffer without imidazole, while gradually decreasing the urea concentration in a stepwise manner (6, 4, 2, 1, and 0 M). After stepwise removal of urea, the column was washed and eluted using standard Ni²^+^-NTA buffer containing imidazole. Finally, the purified fractions were analyzed using SDS-PAGE.

The eluted fractions containing EPC3-ND or All-S mutant were transferred to an ultrafiltration tube (Amicon Ultra, MWCO 10 kDa) and concentrated at 4,900 × g at 4°C using an SRX-201 high-speed refrigerated centrifuge (TOMY). The concentrated protein solution was then transferred to a dialysis membrane tube (Spectra/Por 1, MWCO 6–8 kDa) and sequentially dialyzed against three different dialysis buffers for 4–6 h: (1) 1 mM EDTA, 25 mM Tris-HCl, and 150 mM NaCl, pH 8.0; (2) 25 mM Tris-HCl and 100 mM NaCl, pH 8.0; and (3) 25 mM Tris-HCl and 50 mM NaCl, pH 8.0. The dialyzed soluble fraction was collected, and TEV protease (Sigma-Aldrich, T4455, recombinant from *E. coli*) was added at a ratio of 100 U protease per 2 mg of fusion protein to cleave the cleavage site. The sample was incubated at 30°C with gentle shaking for 19 h. Subsequently, the cleaved target protein was purified using a high-performance liquid chromatography (HPLC) system (SHIMADZU LC-10AD VP) equipped with a COSMOSIL Protein-R packed column (Nacalai Tesque, Kyoto, Japan). The purification was carried out at a flow rate of 0.5 mL/min for 50 min at room temperature using a linear gradient from 20 to 30% of solvent B (0.1% trifluoroacetic acid (TFA) in acetonitrile (CH_3_CN) in solvent A (0.1% TFA in water). The purified fractions were collected and vacuum-dried using an Eppendorf Concentrator plus.

The molecular mass of the purified protein was measured by matrix-assisted laser desorption/ionization time-of-flight mass spectrometry (MALDI-TOF-MS) in the linear mode using a Bruker UltrafleXtreme instrument. The sample was prepared at a concentration of approximately 100 μM. A 1μL aliquot of the sample solution was mixed with 10 μg/μL of sinapinic acid (SA), dissolved in 50% CH_3_CN containing 0.1% TFA. To assess the disulfide pairs in EPC3-ND, the purified sample was subjected to trypsin digestion in 25 mM Tris-HCl buffer (pH 8.0) without reducing agents to retain the disulfide linkages. The resulting peptide fragments were analyzed by MALDI-TOF-MS.

### 2.5 Structural study of EPC3-ND using NMR

Uniformly (^15^N, ^13^C)-labeled EPC3-ND were expressed in *E. coli* using M9 minimal medium supplemented with ^15^NH_4_Cl and ^13^C-glucose as the sole nitrogen and carbon sources, respectively, following the same protocol used for the unlabeled sample. The uniformly (^15^N,^13^C)-labeled EPC3-ND were dissolved in an aqueous solution of 50 mM NaCl and 10% D_2_O (pH 7.0). Nuclear magnetic resonance (NMR) spectra were acquired using a Bruker AVANCE III spectrometer (^1^H Larmor frequency = 800 MHz) equipped with a TCI cryogenic probe. During NMR experiments, the samples were maintained at 308 K. The following spectra were recorded: ^1^H-^15^N HSQC (Kay et al., 1992), HNCA (Kay et al., 1990; Grzesiek and Bax, 1992a), CBCA(CO)NH (Grzesiek and Bax, 1992b), HNCACB (Grzesiek and Bax, 1992c), C(CO)NH (Grzesiek and Bax, 1992b), HNCO (Kay et al., 1990; Grzesiek and Bax, 1992a; Muhandiram and Kay, 1994), ¹H-¹³C CT-HSQC (Hallenga and Lippens, 1995), HCCH-TOCSY (spinlock time = 22 ms) (Kay et al., 1993; Bax et al., 1990), ^15^N-separated TOCSY (spinlock time = 70 ms) (Grzesiek and Bax, 1992a), ¹³C-separated NOESY (mixing time = 120 ms) (Zwahlen et al., 1997), and ^15^N-separated NOESY (mixing time = 120 ms) (Marion et al., 1989).

The resulting free-induction decay (FID) signals were processed using standard NMR data processing methods. Fourier transformation of the acquired data was performed using NMRPipe (Delaglio et al., 1995), and spectral analysis was conducted using Sparky (Goddard and Kneller, 2008). Structural calculations were performed using CYANA ver. 2.0 (Güntert and Buchner, 2015). Structures were visualized using PyMOL (Schrödinger, 2015) or MOLMOL (Koradi et al., 1996).

NMR relaxation experiments were also carried out. The ^15^N relaxation experiments (*T*_1_, *T*_2_, and ^15^N-{^1^H} NOE) were recorded using ^1^H-^15^N HSQC-based pulse sequences (Farrow et al., 1994). The *T*_1_ delays used were 40, 80, 150, 240, 300, 500, and 700 ms, while the *T*_2_ delays were 25, 60, 80, 120, 250, 500, and 700 ms. To assess the backbone dynamics of EPC3-ND, [¹H]-saturation-based heteronuclear NOE (hetNOE) experiments were also performed. Two spectra with and without proton saturation were recorded. Peak intensities were extracted from each 2D spectrum using Sparky (Goddard and Kneller, 2008), following the resonance assignment. The extracted peak intensities were exported and analyzed using Mathematica ver. 12.1 (Wolfram Research Inc.) and the relaxation times were calculated by fitting the intensity decay curves to a single exponential function. The following equation was used for curve fitting:


$$I\left(t\right)=I_{0}\cdot \exp\left(-t/T_{1,2}\right)$$

## 3 Results

### 3.1 Design of the EPC3 fragments

EPC3 consists of 207 amino acid residues. To investigate structural features, we performed secondary and tertiary structure predictions using Jpred4 (Drozdetskiy et al., 2015) and AlphaFold2 (Jumper et al., 2021), respectively. The approximately 50 N-terminal residues of EPC3 were expected to be intrinsically disordered, followed by β-rich elements. In the predicted three-dimensional model, EPC3 was expected to comprise two distinct domains corresponding to residues 44–111 and 112–207, which were folded as the N- and C-terminal globular domains, respectively. These two domains were connected by a short linker, as inferred from the predicted model. Structural comparison suggested a notable similarity to the X-ray crystal structure of the effector protein SIX6 (Yu et al., 2024), although only EPC3 had an N-terminal unstructured region.

**Figure 1:**
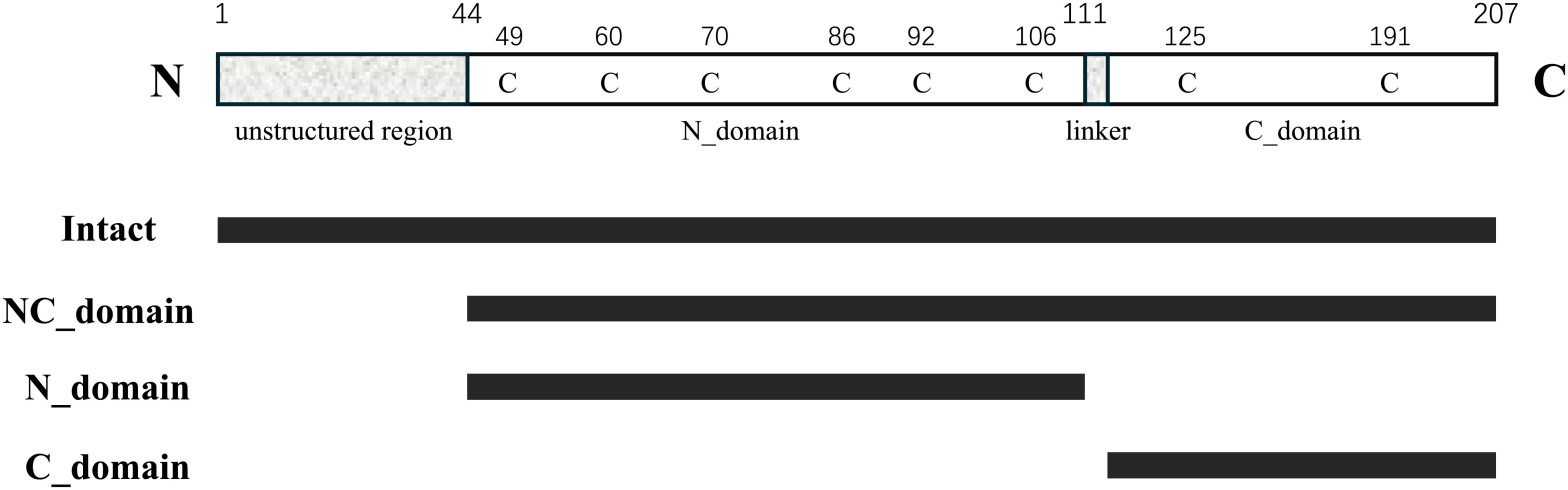
Schematic representation of primary structure of EPC3. In the top, 'C' represents cysteine residue. From second to bottom, each bar indicates the fragment expressed in this work.

To identify the functional roles of the two EPC3 domains, we assessed the activity of full-length EPC3 and its three fragments: the EPC3-N domain (EPC3-ND), EPC3-C domain (EPC3-CD), and EPC3-NC domain (EPC3-NCD). **Figure 1** shows the domain architecture of EPC3, indicating the fragments used in the activity assay.

### 3.2 Activity of EPC3 and its fragments for suppression of flg22-triggered ROS generation

EPC3 is involved in the virulence of *C. orbiculare* in host plants; therefore, we hypothesized that EPC3 possesses the ability to suppress plant immune responses. To assess this, we investigated whether EPC3 can suppress ROS generation triggered by flg22 (Gómez-Gómez and Boller, 2002), a conserved bacterial MAMP, in *Nicotiana benthamiana* via *Agrobacterium*-mediated transient expression assay, as previously reported (Irieda et al., 2019).

**Figure 2:**
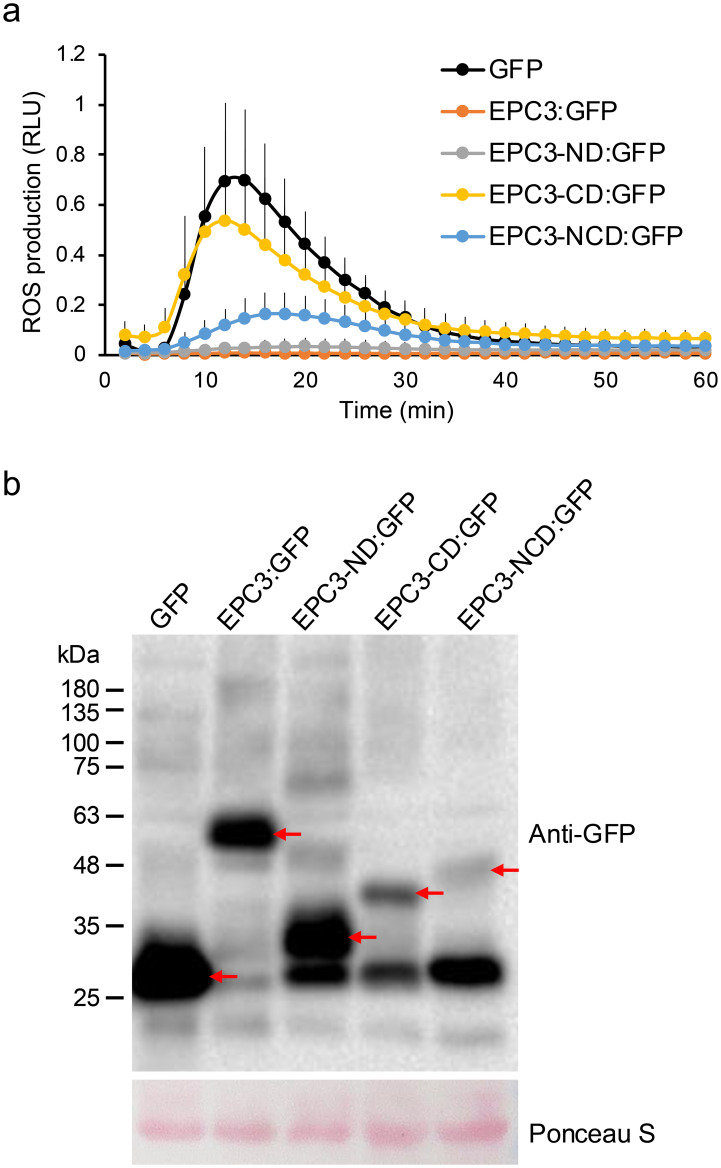
Functional analysis of EPC3 domains. **(A)** EPC3, EPC3-ND, and EPC3- NCD suppressed flg22-triggered ROS production in N. benthamiana. The total ROS production was measured in N. benthamiana transiently expressing EPC3: GFP (EPC3 with signal peptide), EPC3-ND:GFP, EPC3-CD:GFP, EPC3-NCD:GFP and GFP (negative control). Data are presented as mean ± SE (n = 12). Similar results were obtained from two additional experiments. **(B)** Western blot analysis to investigate the protein accumulation of EPC3: GFP, EPC3-ND: GFP, EPC3-CD:GFP, EPC3-NCD: GFP, and GFP transiently expressed in N. benthamiana. Red arrows indicate the corresponding expressed protein bands. Ponceau S staining was used as a loading control.

Treatment with flg22 induced ROS generation in *N. benthamiana* expressing intact GFP alone as a negative control (**Figure 2A**). In contrast, flg22-triggered ROS generation was almost completely abolished when the full-length EPC3 fused to GFP at its C-terminus (EPC3:GFP) was transiently expressed. These results indicated that EPC3 can suppress MAMP-triggered ROS generation, a typical MAMP-triggered plant immune response, in *N. benthamiana.* Thus, we used the flg22-triggered ROS generation assay to evaluate the activity of EPC3 fragments fused to GFP at the C-terminus (**Figure 1**). The flg22-triggered ROS assay revealed that EPC3-CD: GFP did not suppress ROS generation (**Figure 2A**). In contrast, EPC3-ND: GFP largely suppressed ROS generation, similar to EPC3:GFP (**Figure 2A**). Western blot analysis using an anti-GFP antibody revealed that EPC3:GFP, EPC3-ND: GFP, and EPC3-CD: GFP accumulated in *N. benthamiana*, suggesting that EPC3-ND is important for suppressing flg22-triggered ROS generation (**Figure 2B**). Consistent with this finding, we found that EPC3-NCD: GFP partially suppressed ROS generation (**Figure 2A**), although the accumulation of EPC3-NCD: GFP was markedly lower than that of EPC3-CD: GFP (**Figure 2B**). Collectively, these results indicated that EPC3-ND is the functional domain of the effector EPC3 from *C. orbiculare*. Therefore, we performed NMR analysis of EPC3-ND.

### 3.3 Three-dimensional structure of EPC3-ND

Intramolecular disulfide (SS) bond pairs in the recombinant EPC3-ND sample were analyzed before NMR analysis. Matrix-assisted laser desorption/ionization time-of-flight mass spectrometry (MALDI-TOF-MS) confirmed the presence of three intramolecular disulfide bond pairs in the purified EPC3-ND. To identify specific disulfide linkages, we performed enzymatic digestion using trypsin, which cleaves lysine and arginine residues at the C-terminus. The resulting peptide fragments were analyzed by MALDI-TOF-MS. The data shown in **Supplementary Figure S1** clearly indicates that EPC3-ND contains three disulfide bond pairs: Cys49-Cys70, Cys60-Cys92, and Cys86-Cys106, suggesting that there is no free cysteine residue in EPC3-ND.

The ^1^H-^15^N HSQC spectrum of ^15^N-labeled EPC3-ND is shown in **Supplementary Figure S2**. Numerous peaks were observed in the ^1^H-^15^N HSQC spectrum of purified EPC3-ND. The number of peaks was significantly greater than the number of residues (**Supplementary Figure S2A**), suggesting that the purified sample existed in multiple conformational states. Therefore, we recorded a series of ^1^H-^15^N HSQC spectra at different temperatures and/or pH values to determine the optimal conditions under which the molecules form a single conformation. Heat treatment effectively induced protein refolding. The sample was incubated at 90°C for 5 mins and immediately cooled down on ice. No precipitation was observed during the annealing process. In the ^1^H-^15^N HSQC spectrum of the annealed sample, a reasonable number of peaks appeared, as shown in **Supplementary Figure S2B**. In the ^1^H dimension of the ^1^H-^15^N HSQC spectrum, almost all cross-peaks were distributed in the range of 6.5 to 9.5 ppm. The sharp and well-dispersed peaks clearly indicate that EPC3-ND adopts a stable single conformation suitable for further NMR analysis.

**Figure 3:**
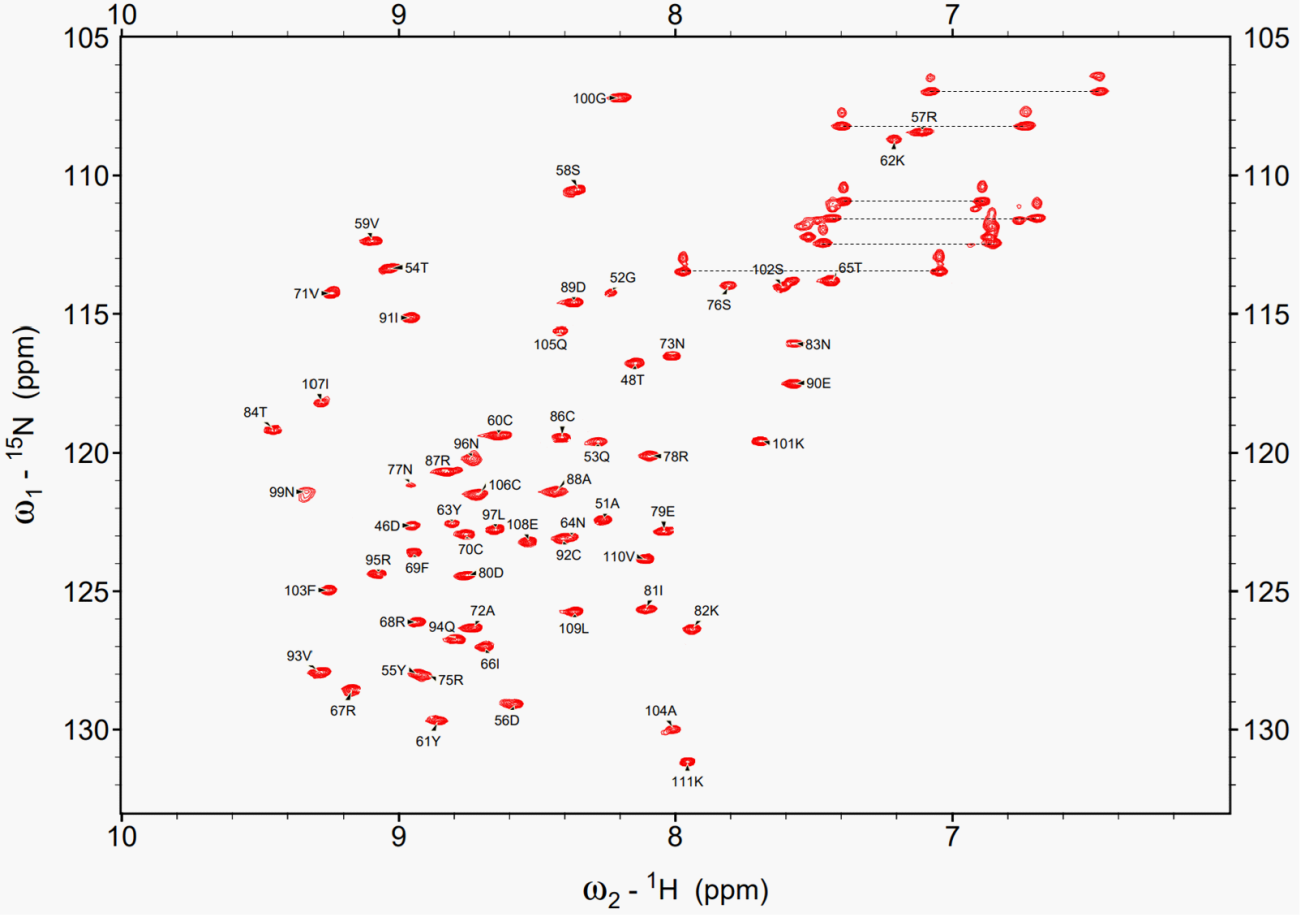
1H-15N HSQC spectrum of EPC3-ND acquired under the optimized condition. All mainchain cross-peaks are fully assigned. Each peak is labeled with the one-letter amino acid code and residue number corresponding to the EPC3-ND sequence.

Under the optimized conditions, a set of triple-resonance NMR experiments, including HNCA, HNCO, HNCACB, and CBCA(CO)NH was recorded for backbone resonance assignments. As a result, 94% of the non-proline backbone amide signals were successfully assigned (64 residues, excluding four Pro residues). The missing signals were at Gly44, Arg47, Cys49, and Ser98. Signals corresponding to these four residues were absent in the ¹H-^15^N HSQC spectrum, suggesting that these residues are located in flexible regions or are involved in conformational exchange whose rate is intermediate on the NMR time scale. **Figure 3** shows the ^1^H-^15^N HSQC spectrum acquired under optimized conditions, with assignments of all observed backbone resonances. For side-chain resonance assignments, we recorded 2D- and 3D-NMR data including ^1^H-^13^C CT-HSQC, C(CO)NH, HCCH-TOCSY, and ^15^N-separated TOCSY. Side-chain carbon resonances were assigned to 66 of 68 residues, with only Gly44 and Cys49 unassigned, resulting in slightly higher coverage than that of the backbone. Analysis of the ¹³Cβ chemical shifts of the cysteine residues, except Cys49, revealed that all had values above 35 ppm, the chemical shift range typically associated with oxidized cysteines involved in disulfide bond formation (Sharma and Rajarathnam, 2000). This observation was consistent with the MS results, which indicated that all Cys residues were in the oxidized form.

**Figure 4:**
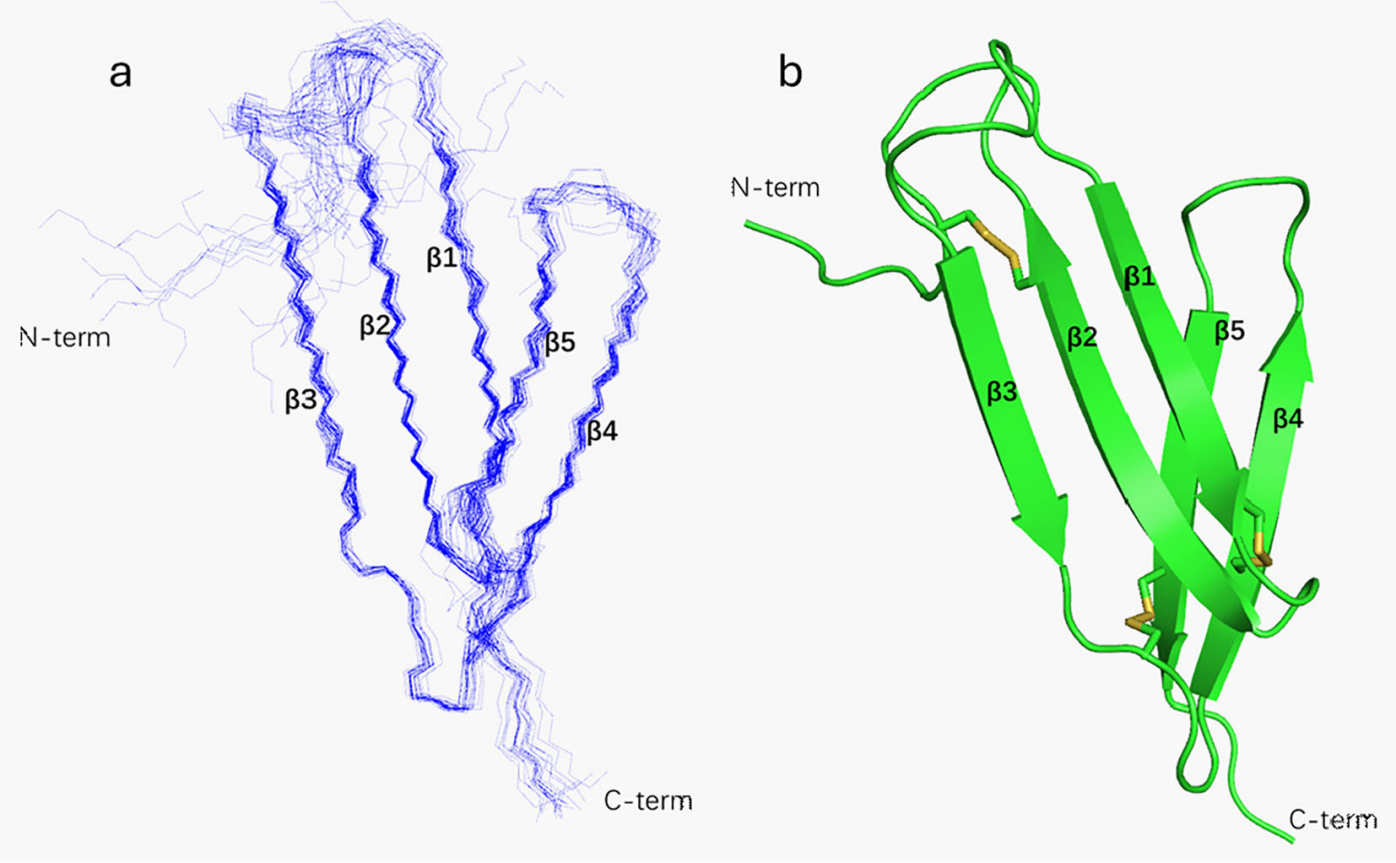
NMR solution structure of EPC3-ND. **(A)** Superposition of the 20 lowest-energy conformers of EPC3-ND are depicted with wire model. **(B)** Ribbon diagram of EPC3-ND with SS-bonds (yellow). Well-defined B-strands are named as B1 to B5 from the N-terminal end.

After resonance assignment, the NOE peaks were collected. Subsequently, calculations based on CYANA were performed using NOE-derived distance restraints. The dihedral angle constraints estimated using TALOS were employed in the structural calculations. Hydrogen bond restraints (38 in total) for the secondary structural regions were introduced based on the characteristic NOE patterns and preliminary structural information. In addition, distance restraints corresponding to the three disulfide bonds determined by MS analysis were incorporated. The resulting ensemble of the 20 lowest-energy conformers is shown in **Figure 4A**, representing the converged solution structure of EPC3-ND. The root-mean-square deviation (r.m.s.d.) calculated for Cα atoms across all residues is 1.926 Å. The structural statistics are summarized in **Supplementary Table S2**. EPC3-ND has five β-strands encompassing residues Tyr55-Tyr61 (β1), Asn64-Val71 (β2), Glu79-Thr84 (β3), Glu90-Asn96 (β4), and Ser102-Glu108 (β5). They form two β-sheets, comprising two and three strands, respectively. The two sheets are packed against each other and are interconnected through three disulfide bonds that bridge the N-terminal loop to β2, β1 to β4, and the loop following β3 to β5 (**Figure 4B**). Thus, the covalent linkage formed by these disulfide bonds is thought to be a key factor in stabilizing the overall conformation. Their contribution to the structural stability was examined using an EPC3-ND mutant lacking disulfide bonds. The results are presented in the next section.

### 3.4 Backbone dynamics and structural stability of EPC3-ND

**Figure 5:**
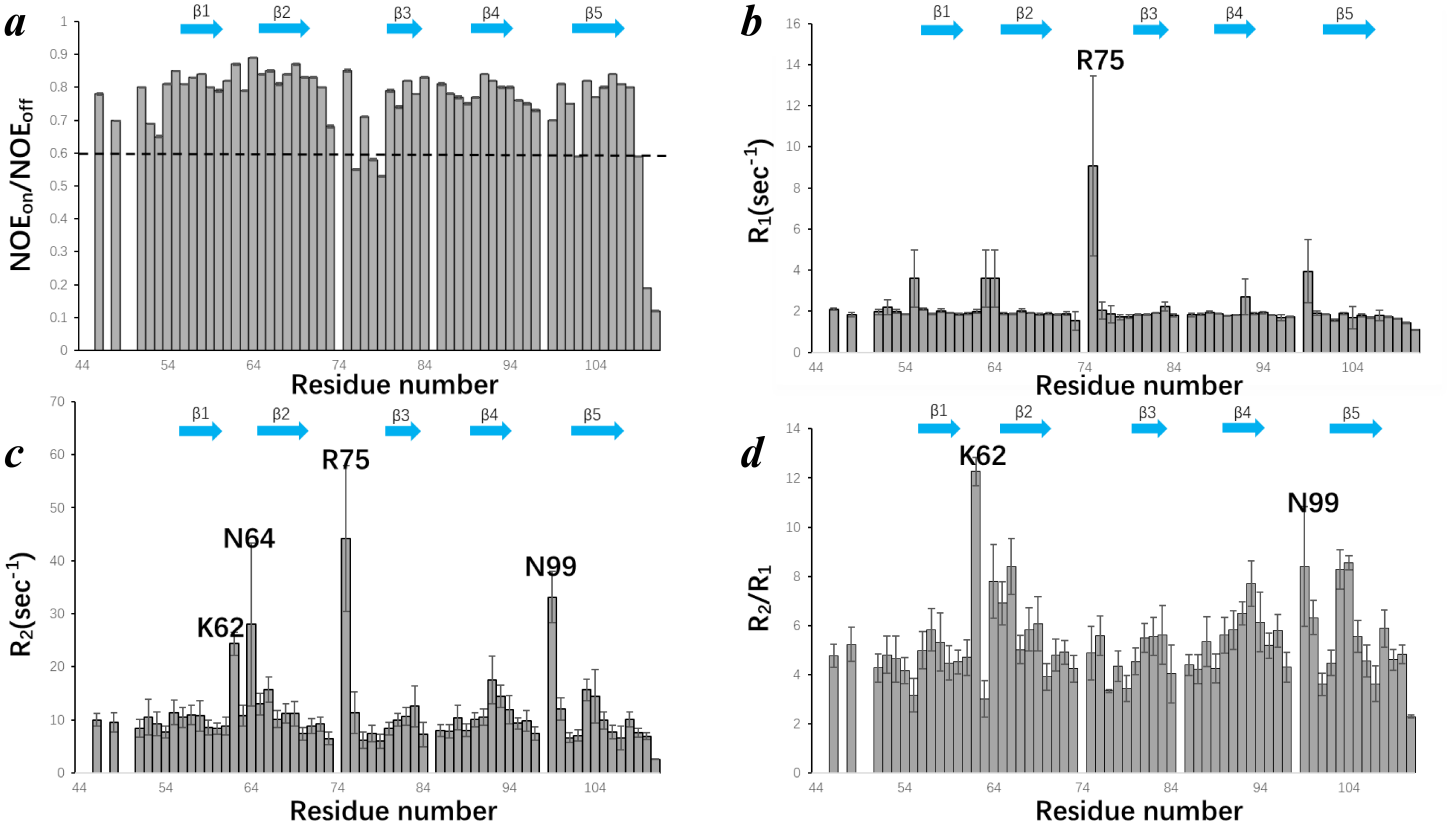
Backbone dynamics of EPC3-ND assessed by solution NMR relaxation experiments. **(A)** Steady-state heteronuclear {H}-15N NOE values plotted as a function of residue number. The dashed line at NOE = 0.6 marks the threshold commonly used to distinguish rigid and flexible regions. **(B)** Longitudinal relaxation rates (R) of each residue. **(C)** Transverse relaxation rates (R2) of each residue. **(D)** R2/R: ratios across the EPC3-ND sequence. In each panel, B-strand regions are shown by arrow. Some residues showing distinguish values are labeled with amino acid type and residue number.

Next, we performed NMR relaxation experiments to reveal the internal dynamics of EPC3-ND. We measured the steady-state heteronuclear NOE, longitudinal relaxation rates (*T*_1_), and transverse relaxation rates (*T*_2_). The results are shown in **Figure 5**.

**Figure 5A** shows the ^15^N-{^1^H} heteronuclear NOE values (NOE_on_/NOE_off_) plotted against residue numbers. The NOE value of 0.6 (indicated by a dashed line) is generally considered a threshold for backbone flexibility; values above 0.6 suggest rigid backbone conformations, while those below indicate increased local flexibility. The NOE values for EPC3-ND are consistently high across the β-strand regions (β1-β5), reflecting a well-ordered β-sheet core. In contrast, the residues near the terminal and loop regions displayed reduced NOE values, indicating enhanced local flexibility. **Figure 5B** presents the *R*_1_ (=1/*T*_1_) relaxation rates, which reflect fast (picosecond-to-nanosecond) timescale motions. Most residues show similar *R*_1_ values around 2 Hz, consistent with a structured protein. Notably, Arg75 displayed a pronounced value, suggesting elevated internal motion or potential conformational exchange. Interestingly, Arg75 is located in the longest loop region connecting β2 and β3. **Figure 5C** depicts the *R*_2_ (=1/*T*_2_) relaxation rates. While most residues maintain moderate *R*_2_ values, residues Lys62, Asn64, Arg75, and Asn99 exhibit elevated *R*_2_ rates with relatively large error ranges, which considerably reflect microsecond-to-millisecond timescale motions or conformational exchange processes. These residues are located in loop or solvent-exposed regions, contrasting with the more rigid β-strand elements. Notably, the large *R*_2_ value for Asn99 was related to the missing Ser98 signal in ^1^H-^15^N HSQC, suggesting that this region has some motion. **Figure 5D** shows the *R*_2_/*R*_1_ ratio for each residue, which serves as a useful indicator of chemical exchange and/or motions on intermediate timescales. The *R*_2_/*R*_1_ ratios of Lys62 and Asn99 further support the presence of conformational dynamics or exchange processes in these regions. These observations are consistent with the flexible loop architecture and possibly imply functional plasticity or involvement of intramolecular interactions. In summary, these relaxation data provide comprehensive insights into the residue-specific internal dynamics of EPC3-ND. The results highlight the rigidity of the β-sheet core and the dynamic nature of loop regions, particularly those centered around Lys62, Arg75, and Asn99.

To assess the contribution of intramolecular disulfide bonds to the conformation of EPC3-ND, we prepared a variant, All-S, in which all six cysteine residues were substituted with serine. Initial attempts to purify the All-S protein by ultrasonic disruption resulted in apparent degradation, as evidenced by the appearance of multiple lower-molecular-weight fragments on SDS-PAGE (**Supplementary Figure S3A**). This degradation was likely due to structural disruption during sonication, although the possibility of limited proteolysis by endogenous *E. coli* enzymes cannot be completely excluded. To mitigate the mechanical denaturation of the tagged All-S sample, we adopted a mild lysis approach using lysozymes. Under these conditions, the SDS-PAGE profile showed no significant degradation in the molecular weight (**Supplementary Figure S3B**), enabling successful purification of the All-S protein. However, ^1^H-^15^N HSQC of the purified All-S sample revealed a severe loss of chemical shift dispersion, which was consistent with an unfolded state (**Supplementary Figure S4**). The small number of observed peaks and broadened line shapes also suggest that the sample molecules aggregated in the NMR tube. Even after applying heat treatment to the All-S sample, the ^1^H-^15^N HSQC spectrum showed no marked improvement in chemical shift dispersion. This indicates that, in the absence of disulfide bonds, the unfolded and aggregated state of EPC3-ND is irreversible. These findings underscore the essential role of disulfide bonds in maintaining the structural integrity of EPC3-ND. The absence of intramolecular disulfide bonds led to loss of the defined tertiary structure, highlighting the disulfide-dependent nature of EPC3-ND folding. These findings, combined with the rigidity observed in β-strand regions, indicate that the disulfide bonds contribute to forming a highly restricted and thermodynamically stabilized conformation with a minimum energy level in the folding landscape. Due to the lack of information regarding the interacting partner(s), it remains unclear whether the disulfide bonds of EPC3-ND are directly involved in the target binding site. However, these disulfide bonds are buried and unexposed on the molecular surface, they are more likely to play a structural role in maintaining the overall conformation of EPC3-ND, which is required for molecular recognition.

## 4 Discussion

### 4.1 The N-terminal half of EPC3 is responsible for its function

EPC3 of *C. orbiculare* has been identified as a virulence effector for pathogen infection in cucurbitaceous host plants (cucumbers and melons) (Inoue et al., 2023). In this study, we revealed that EPC3 suppressed flg22-triggered ROS generation in *N. benthamiana.* Because we found that EPC3:GFP suppressed ROS generation, we considered EPC3:GFP to be functional, at least when transiently expressed in *N. benthamiana* cells. Our previous report revealed that EPC3 is not essential for infection in *N. benthamiana* by *C. orbiculare* (Inoue et al., 2023). However, we found that EPC3 suppressed the immune response in *N. benthamiana.* Thus, EPC3 is specifically required for cucurbit infection; however, EPC3 is likely to be functional in other plants, such as *N. benthamiana*, in addition to its cucurbitaceous hosts.

Based on the secondary and tertiary structure predictions of EPC3, we identified two distinct domains in EPC3, designated EPC3-ND and EPC3-CD. Importantly, we found that EPC3-ND, but not EPC3-CD, was essential for suppressing flg22-triggered ROS generation, indicating that EPC3-ND is the functional domain of the effector EPC3. In addition, ROS suppression by EPC3-NCD cells was lower than that by EPC3-ND cells. This is likely due to the lower accumulation of EPC3-NCD. Although EPC3-ND lacks the signal peptide of EPC3, it suppresses ROS generation when transiently expressed. This finding suggests that EPC3 functions as a cytoplasmic effector in plant cells, although further localization analyses are necessary to draw a definitive conclusion.

### 4.2 Structural comparison with homologous proteins

**Figure 6:**
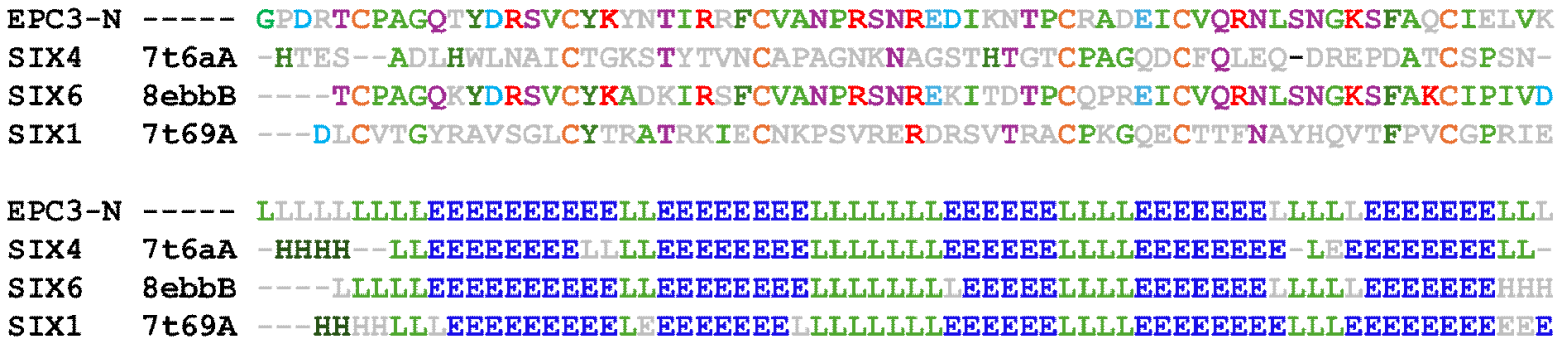
Sequence and secondary structure alignment of EPC3-ND with *Fusarium oxyporum* SIX effectors. In the top panel, amino acid sequence alignment is shown. Conserved and functionally relevant residues are highlighted in color based on residue type. The lower panel illustrates predicted secondary structure elements, where β-strands (E), α-helices (H), and loops/coils (L) are indicated. For the SIX family effectors, PDB codes are alos shown in the figure.

To investigate the structural similarity of EPC3-ND with known effector proteins, we conducted a structural homology search using the DALI server. The results revealed a moderate structural resemblance between EPC3-ND and members of the SIX effector family, particularly SIX1, SIX4, and SIX6, which are secreted by *Fusarium oxysporum* and play roles in host-pathogen interactions. Among these, the N-terminal domain of SIX6 showed the highest structural similarity with that of EPC3-ND (Z-score = 3.8). The backbone Cα atom r.m.s.d. between EPC3-ND and the N-terminal domain of SIX6 is 2.083 Å, suggesting a potentially conserved β-sandwich fold shared among these effectors. Although their core β-sheet architecture is well conserved, they show limited sequence identity (**Figure 6**).

**Figure 7:**
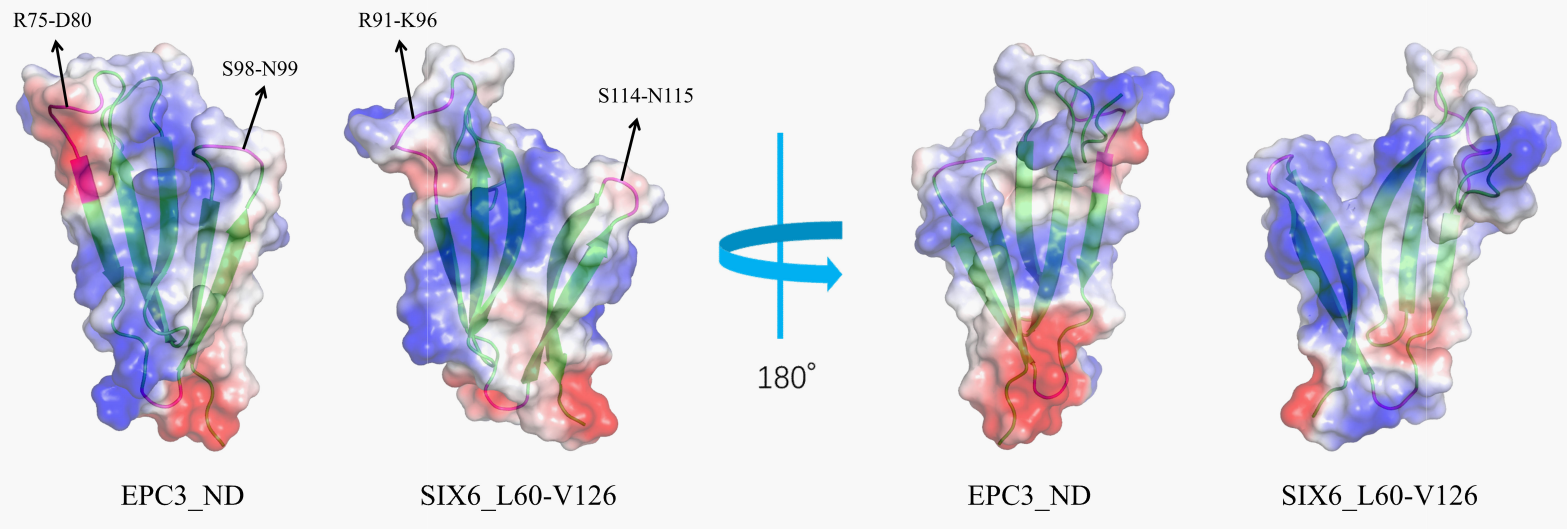
Eloctrostatic surface potentials of EPC3-ND (left) and SIX6 (right) shown in two orientation. Negative, positive and hydrophobic molecular surface is colored with red, blue, and white, respectively. Ribbon model is shown in the molecular with greem color.

Because structural conservation may provide a basis for functional convergence among these effectors via key residues, we analyzed the surface potential of EPC3-ND and the N-terminal domain of SIX6. The electrostatic surface potential of EPC3-ND displays a distribution pattern similar to that of the SIX6 N-domain (Sánchez-Vallet et al., 2013; Yu et al., 2024), featuring a large positively charged region (left in **Figure 7**). Different electrostatic properties were observed in the bottom part of the two molecules, as shown in the right panel of **Figure 7**, indicating that EPC3-ND is negative, whereas SIX6 is positive.

### 4.3 Prediction of functional site and function

Specifically, as shown in **Figure 7** (left), similar structural features and electrostatic potentials were found at sequential residues Ser98-Asn99 in EPC3-ND corresponding to Ser114-Asn115 in SIX6. Moreover, the charged segment Arg75-Asp80 in EPC3-ND corresponds to Arg91-Lys96 in SIX6, forming a surface-exposed patch with comparable electrostatic characteristics. Notably, the Ser98-Asn99 region has conformational flexibility, as suggested by the relaxation experiments. Similarly, residues Arg75-Glu79 of EPC3-ND display increased backbone flexibility, as indicated by reduced ^15^N-{^1^H} NOE values (<0.6). These features highlight the dynamically active and surface-exposed regions that may contribute to molecular recognition. These structural and dynamic features imply that EPC3-ND and SIX6 share similar target-recognition modes. Although further investigations are required to confirm the functional involvement of these residues, our observations provide a structural basis for future mutational and binding studies.

Previous studies have indicated that the N-domain of SIX6 shares structural similarity with known chitin-binding proteins such as hevein and tachycitin (Beintema, 1994; Yu et al., 2024). These proteins possess conserved aromatic residues (e.g., tryptophan and tyrosine) on their surfaces, which are crucial for carbohydrate binding (Van Damme et al., 2008). Since EPC3-ND and SIX6 lack such aromatic side chains on their molecular surfaces, it is possible that if they mediate similar carbohydrate recognition, the interaction may rely on electrostatic or hydrogen-bonding mechanisms rather than classical aromatic stacking.

### 4.4 Concluding remarks

In summary, we identified the N-domain as the functional core of EPC3. Structural analysis revealed that this domain adopts a β-sandwich fold composed of five β-strands. NMR relaxation analysis revealed dynamic motion in certain residues that may be involved in target binding, although the identity of the binding target remains unknown. Biochemical validation of the functional similarity between EPC3 and SIX6 may also provide insights into the role of the N-terminal extension. Further functional and structural investigations, particularly those focusing on the C domain, are required to fully elucidate the mechanism of the intact EPC3 effector.

## Data Availability

The datasets presented in this study can be found in online repositories. The names of the repository/repositories and accession number(s) can be found below: http://www.wwpdb.org/, 9W4X.
