# Supplementary figures and images for: Identification and NMR-based structural characterization of the functional domain of EPC3, a virulence effector of the phytopathogenic fungus Colletotrichum orbiculare
Source: Front Plant Sci. 2025 Oct 10;16:1691993. doi: 10.3389/fpls.2025.1691993 (PMC12549616; doi:10.3389/fpls.2025.1691993)

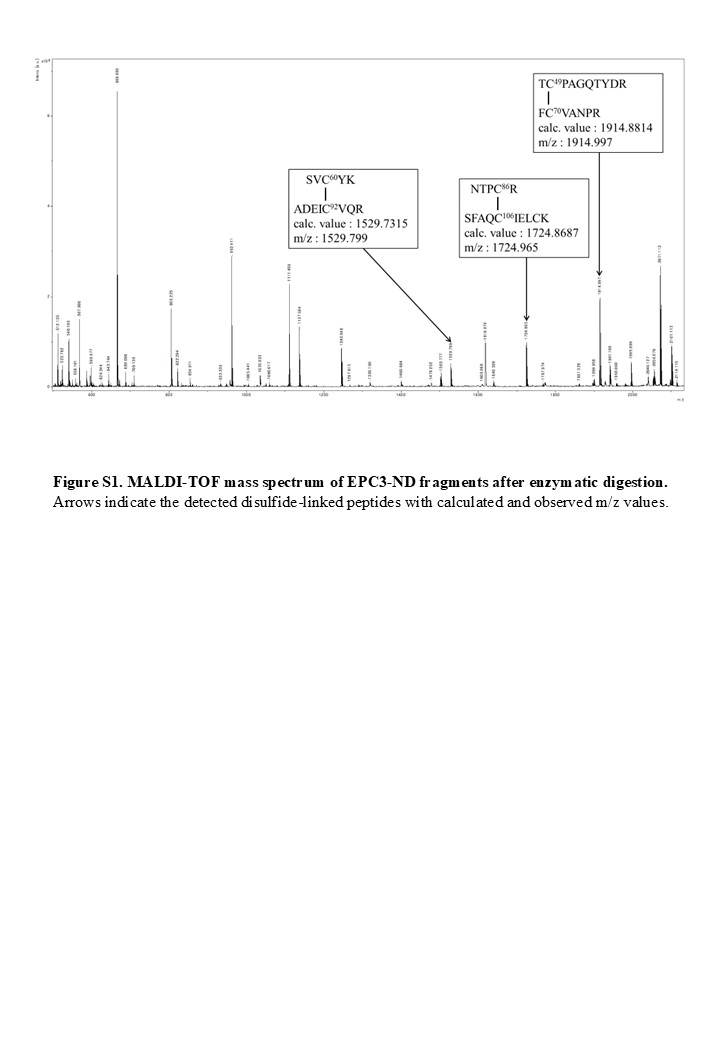

Supplement: Supplementary file 1 [file Image1.jpeg]

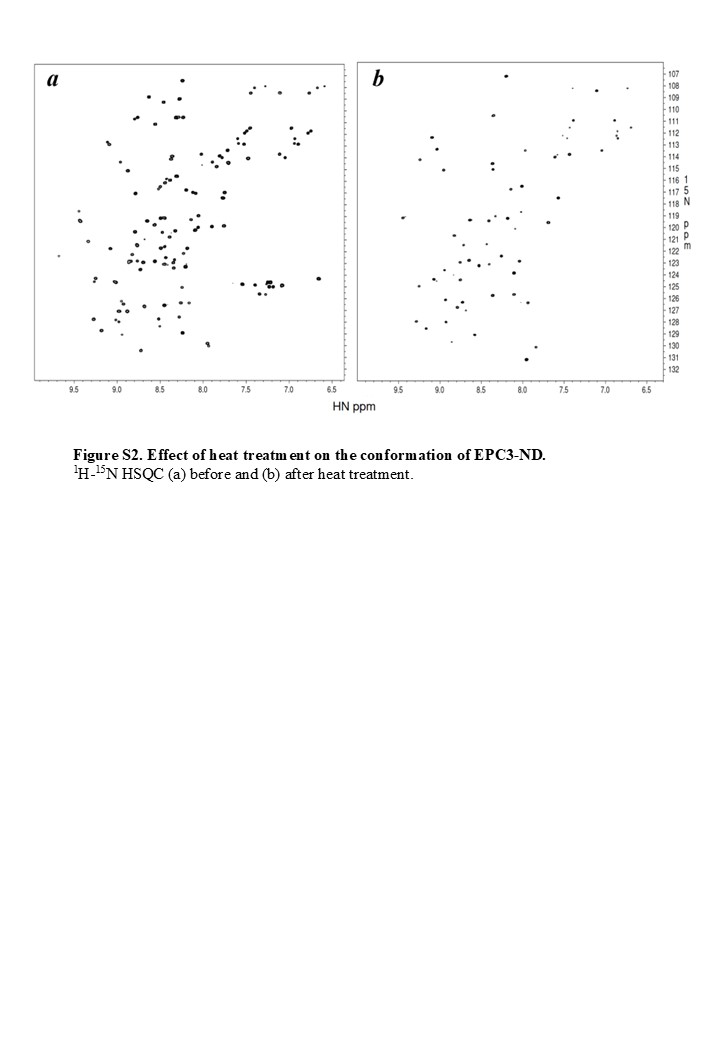

Supplement: Supplementary file 2 [file Image2.jpeg]

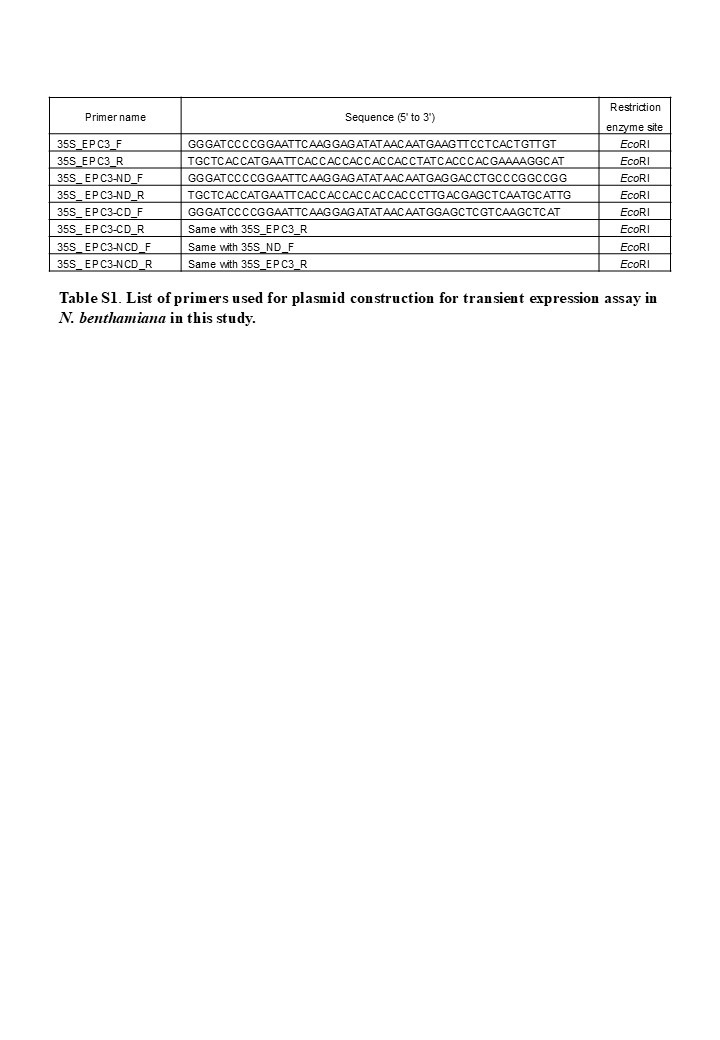

Supplement: Supplementary file 3 [file Image3.jpeg]

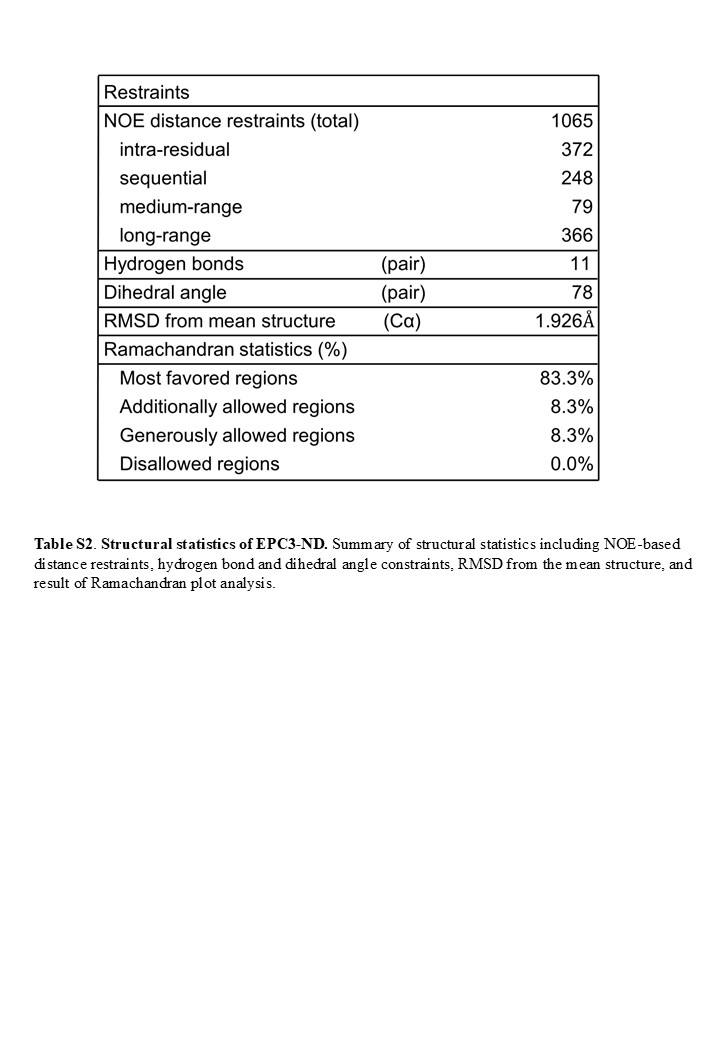

Supplement: Supplementary file 4 [file Image4.jpeg]

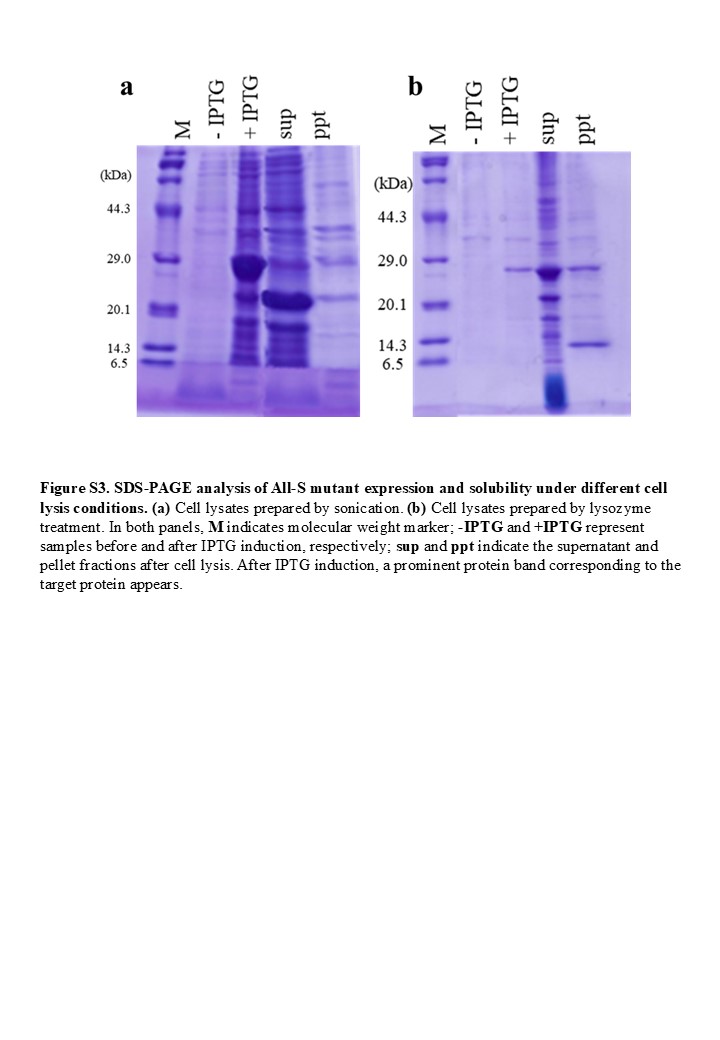

Supplement: Supplementary file 5 [file Image5.jpeg]

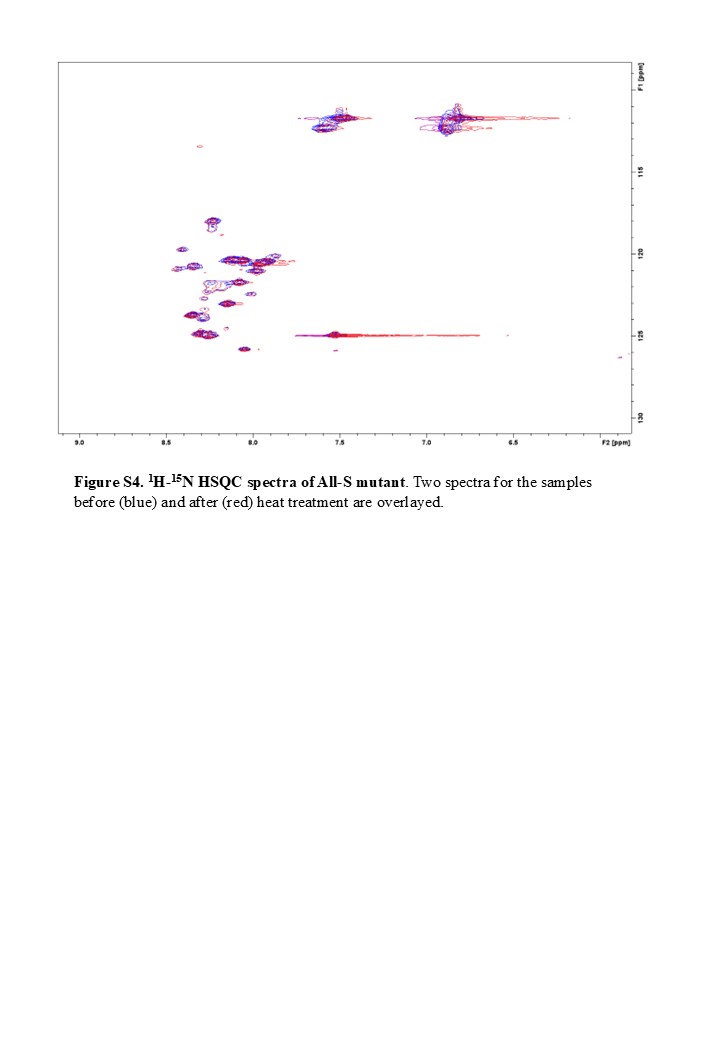

Supplement: Supplementary file 6 [file Image6.jpeg]
